# Medical Jurisprudence, Chemistry and Psychology

**Published:** 1877-07

**Authors:** D. R. Brower


### SECTION IV.—MEDICAL JURISPRUDENCE, CHEMISTRY AND PSYCHOLOGY.

Reported by D. R. Brower, M. D.

First Day—Tuesday, June 5th.

Dr. Eugene Grissom, of North Carolina, Chairman.

Dr. E. A. Hildreth. of West Virginia, Secretary.

The first paper read was entitled

RELATIONS OF SPIRITUALISM TO MEDICAL JURISPRUDENCE, by John P. Gray, M. D., LL. D., Sup't New York Lunatic Asylum, Utica, N. Y.

It was a paper exhibiting painstaking in its preparation, excellent judgment in its deductions, and reflecting great credit on its able author. The doctor stated that spiritualism is not a form of religious belief in any sense, ordinary or theological, but embraces persons belonging to all religious denominations, as well as those who do not believe in divine revelations; that it has led many persons into practices at variance with sound morality; has led persons to disregard the principles of common statute law in regard to business matters, to giving away their property; that wills, contracts and speculations have been made under direction of so-called spirits and spirit communications; and that such acts have brought the subject at least indirectly within the scope of medico-legal investigation; moreover, many of the phases of human conduct in these people might represent moral perversion or folly, religious or social, and therefore appear to belong to the moralist or theologian than to the physician or lawyer, and might be classed among erratic or superstitious ideas.

Dr. G. then in illustration detailed the case of Capt. E. B. Ward, tried in Detroit, in 1876. In this case it was proposed to set aside a will, on the plea of insanity. It was asserted that there had been an entire change of Capt. B.'s character that was the outgrowth of cerebral disease, and various acts fully sustained this assertion. The court ruled that spiritualism in any of its manifestations, or so-called phenomena, could not be claimed as tending to show the probability of insanity, but did admit it as a plea of undue influence and that a delusion incident thereto was not an insane delusion. Dr. G. maintained that the court had gone too far, when it placed the communications from spirits on a par with those from living persons, and that there was a vast difference in the influence that would be exerted by communications from living persons and the so-called communications from another world, and referred in this connection to the superstitions of the past, so-called moral epidemics and witchcraft, vampirism, etc. That any one could recognize this principle, and that these mediums undoubtedly appreciated the mingled emotions of terror, curiosity, and religious superstition and brought them into active play in their performances. This can be seen even in the vague terror of a child who hears a strange sound, in the dark, and in the more intelligent fear of the same child when he meets a ferocious animal; the difference being that, between seeing and understanding a danger and so in a measure preparing for it, and that of losing, on the other hand, all control in the midst of dread unknown. This distinction must have weight touching the question of undue influence, and the question of the probable development of insanity. He referred to the case of Robert Dale Owen as showing the power of such belief and the consequences of a revulsion.

Capt. Ward was a man of large wealth and extensive business interests, residing in Detroit, Mich., a man of great energy and very aggressive in character; he did not indulge in spirituous liquors or tobacco, but was largely given to sexual excesses; he had no religious belief, but for a number of years was a spiritualist of a fanatical type. He was married and had several children. At about fifty years of age, he had a slight cerebral hemorrhage, and soon thereafter procured a divorce by his own exertion, from his wife, a paralytic invalid, and to his own disgrace, he then married a young and voluptuous woman, whom he took into the same house to live with his abused wife. By this woman he had two children; about this time his interest in spiritualism became much intensified; he employed female mediums; kept them about him and through them consulted daily the so-called spirits of dead business men. He fully believed that a medium could describe a person unknown to him by a lock of hair, or by placing a letter of the unknown person against the forehead—a process called psychometrizing; he believed that a certain medium painted portraits while blindfolded, of persons unknown to them; had such pictures painted for himself, and exhibited them to visitors as a proof of spiritualism; he believed in so-called spiritual photographs; he believed in the materialized forms of spirits; he had felt his dead wife strike him a blow; he had shaken hands in the air with an old friend and conversed with him through a medium about the testamentary disposition of his property.

After the death of his wife, which occurred after the birth of the children of the second woman he had taken, and on spiritual communication from her, he framed a will distributing his property equally to the children of both women. He subsequently, at the suggestion of the living wife consulted through two mediums the spirit of her father, and as a result, made a will leaving the bulk of his property to this second woman and her two children, providing a limited annuity to the surviving children of his first wife, disinheriting one of his children because the spirits informed him it was illegitimate; this will allowed the executors ten thousand dollars a year for their services, while the aggregate of annuities for his children was only twelve thousand dollars; it moreover, permitted the executors to appoint one of their number guardian for these children; it required them to give no security, and authorized them to fill vacancies by their own selection.

Capt. Ward belonged to a family with the heredity of insanity—one of his children was insane, and another was imbecile. He had only a limited education, and acquired by his own exertion the vast property he possessed, amounting to several millions of dollars. He died at the age of sixty-three of a stroke of apoplexy.

Dr. Gray's general conclusions on the subject were as follows: Spiritualism cannot be taken as an evidence of insanity. Belief in communications from the unseen world, whether from friends of the dead or other ghostly messengers, is not in itself an insane delusion. The belief that so-called mediums can communicate with the dead has no foundation, and no evidence has yet been presented of the truth of such communications having been made. They all stand simply on the assertions of so-called mediums.

The implication of fraud must stand against all such professed communications, as the dead party cannot be reached except through the consent or power of the so-called medium, and as the living party to whom the communication is made has no power himself of communicating with the spirit. The whole is received simply through the medium. Such communications of third parties cannot be received in courts of law, and are excluded by the rule of rejecting conversations not held in the presence of both parties. If spiritualism is espoused as the result of disease of the brain, being before repugnant to the belief and mental operations of the individual, then it is an insane delusion. Spiritualism, or its so-called communications, must be received simply under such ruling of the courts as undue influence or as fraudulent, or conspiring influences in the case of wills or contracts; and wills and contracts made under such spiritual directions and influences through mediums should be void.

The most serious question would arise where a person should attempt to commit homicide under the direction of the so-called spirits. The presence of a medium in such a case would suggest fraud and conspiracy. If the individual was a spiritualist through life and before the crime was committed, no insane delusion can be claimed unless it can be found in the existence of brain disease. He would have to stand, in that case, upon the same platform as ordinary criminals. Spiritualism can only be considered as an occasional delusion in cases of insanity, and not as a cause or form of true alienation. It stands on the same footing with its progenitors, witchcraft, vampirism, sooth-saying, fortune-telling, etc. Its medico-legal bearing must be determined by the facts in each case, as to whether it is an insane delusion or not,—that is, the offspring of disease of the brain; or simply entertained as a speculative belief with reference to the unseen world, or the possible condition of men after death. Medical science can take no cognizance of it as a speculation more than it can of any other "ism."

Drs. Yemans, Bukham and Compton discussed the paper endorsing its general conclusions, and commending the author for his labor and success in its preparation.

The next paper read was by Dr. E. Seguin, of New York, on the intervention of physicians in education. Dr. S. recommended the intervention of physicians in two ways. First by transferring as much as possible the teaching to the open air, by creating garden schools. This would relieve the schools from the contagious ferments generated by crowding, and diminish the chances of sickness and mortality; at the same time the pupils would learn from nature what they see imperfectly represented in books, and gain by this contact a love of nature and naturalness which would react on their future avocations. These garden schools would contain, not only classes of botany, natural history, etc., but classes of drawing, carving and modeling from nature, whence would issue generations of true artists and superior artisans. The second and most important part of the physician in education ought to be that of a keeper of the balance of the vital forces of the children. He would register the idiosyncracies of the children; measure the difference of build and of capacity of both sides of the body, and of all the double organs, and recommend accordingly certain forms of active or passive exercises, etc. The accommodation of the ears and eyes must be tested in order that each child occupy at school precisely the place, and use the printed type corresponding to his power of vision and audition. But above all, the physician in charge of the school must register the movements of the great vital functions—the pulse, the respiration, and the temperature before and after various studies—in order to establish in figures the measure of a child's capacity for study, beyond which dangerous consequences to physical equilibrium may ensue.

The paper of Dr. E. Seguin was full of valuable suggestions. It elicited no special discussion.

The next paper presented was by Dr. R. J. Patterson, Superintendent of the Hospital for the Insane, Batavia, Ill., entitled, DO FACTS JUSTIFY THE RECOGNITION OF MORAL INSANITY AS A DISTINCT FORM OF DISEASE? To this question, Dr. Patterson replied negatively.

The illustrious Pinel, but more especially Prichard and Ray, have taught that facts do justify the recognition of moral insanity as a distinct form of disease. By these authors, this form of disease is defined to be " an insanity of the affections, and it consists in a perversion of the affections, emotions and passions, and it may show itself in morbid love or hatred, or fear or dread, in sadness or exaltation, or depression of the feelings, without delusion or any appreciable mental aberration, or intellectual impairment." (Ray and Prichard.)

Those who have had much to do with the insane, occasionally see cases of insanity in which the morbid emotional phenomena predominated, especially as signs premonitory to a more pronounced state of intellectual impairment.

They often see cases in which, from trophic or other brain disturbances, the changes in the moral or emotional manifesttations are more prominent than in the intellectual. But it is believed that in all these cases, there may be seen a morbid condition, not only of the affective powers, but also of knowing and reasoning powers. It is argued that the more demonstrative emotional manifestations, are not so distinctive nor of so frequent occurrence, nor so persistent, nor so uncomplicated with morbid intellectual manifestations, as to justify a recognition of them, as a distinct form of insanity. It is claimed that no pathologist is able to say that in all the so-called cases of moral insanity, there are not such changes in the cerebral structures, as to weaken their inhibitory or their propelling forces. None of the cases that have been fully written and adjudicated, are free from well-grounded suspicions of intellectual aberration. Therefore, the term, moral insanity, is not warranted by facts.

Insanity is regarded as a disease of the brain, affecting both the intellectual and emotional faculties. All the forms and shades of insanity depend upon some disease, or abnormal condition of the brain, and the report does not concede that with a healthy brain, any type of insanity exists. Known facts do not justify the opinion that the brain can be so impaired in its powers as to let fly the emotions and passions with irresistible force, while the will power, the knowing and reasoning faculties are sound or in full force.

The author of the report quotes from Ray's medical jurisprudence, as follows: "An appetite or an affection craves and seeks its gratification under the control and guidance of the reflective powers. If the efficiency of the latter has been impaired by disease or congenital defect, that check is not exerted and the person goes along, like a ship drifting without a rudder, at the mercy of the winds and waves. If the force of an affection or an appetite is increased by excitement of disease, then the check of the higher powers is insufficient for the purpose. It is a matter of relative power and it is quite immaterial whether the result proceeds from impaired intellect or irresistible activity of the affective powers." Dr. Ray thus admits that the higher or intellectual powers are given to guide and control, and that the moral powers naturally seek their guidance and control.

Dr. Patterson in his paper maintains that the reason why the intellectual powers fail to fulfil their office of control is, because the brain has been so invaded by diseases as to rob the intellect through its defective instrument of its power to command. Again, Dr. Ray substantially says: "The higher or intellectual powers when in force and health may be overcome by the lesser, when the lesser are excited and increased in force by disease." Thus moral insanity it is said may result.

Dr. Patterson admits that the intellectual powers may be weakened by disease so as to be easily overcome, but denies that the affective powers are even increased in force by disease; and asserts that one of the distinguishing features of all the forms of mania, is excitement with diminished force.

In the reports of 27 American institutions for the insane, into which 47,174 patients were admitted, twenty-two only are classed as moral insanity.

Among the objections assigned in the report, to recognizing moral insanity as a distinct form of disease are:

I. There are probably, at the present day, no cases of insanity in which it can be shown that, as a result of brain disease, the person is a moral maniac, with a *rational intellect;* and if there could be such cases, the number must be insignificant.

II. No human interests can suffer by not recognizing moral insanity as a distinct form of disease. It is not possible that any really insane person will suffer under the influence of expressed testimony; if the opinion shall be insanity simply, without the prefix. The law does not require medical men to classify, and the pathological sections in classification are too numerous for medico-legal purposes or the ends of justice.

III. The term not only does no good, but positive harm, by enabling unscrupulous lawyers to set up a specious plea, simply because nothing else will serve their purpose. Especially is this true in cases of homicide, where the intellect is believed to be rational. Such, for example, are the cases of Sickles, Cole, McFarland and Laura Fair.

IV. Another objection to the recognition of so-called moral insanity, is, that it does not, and probably never can, receive the sanction of our higher courts and most learned lawyers; and further, with the medical superintendents of American institutions for the insane, as shown by the statistics already cited, this form of disease has not of late years, found favor except with a comparative few.

It has never been sanctioned by many of the ablest authors in the field of medical jurisprudence; and the plea of moral insanity wherever made is looked upon with suspicion by a majority of men of learning in the professions, who by special study are most entitled to hold opinions in regard to it.

The discussion was opened by Dr. Gray, of New York. The doctor thoroughly agreed with the sentiments of the paper, but thought Dr. Patterson, in using the numerical method, should take the whole number of insane treated by the successive superintendents through a series of years in the various institutions, instead of taking the entire number in some cases, and only a single year in others, that Dr. P. had quoted the State Lunatic Asylum at Utica for one year, and as among the institutions where no moral insanity had been reported, this was not consistent with facts. Dr. Gray, in the twenty-five years of his superintendency of the institution, had reported no cases of moral insanity in ten thousand insane, while Dr. Brigham, his immediate predecessor in the five years of his superintendency, aggregating about two thousand cases, had reported more cases of moral insanity than Dr. Patterson had presented from all the institutions of the country. Dr. Brigham undoubtedly represented the prevailing views of that period, in recognizing this so-called form —recognizing several classes of moral mania which we now regard simply as single manifestations of cases of general insanity. Dr. Gray did not recognize impulsive or transitory mania as a disease that could come and go in a moment; such would be in conflict with all ideas of physiology and pathology. He recognized no disease of the mind, *per se*, but simply disease of the brain disturbing the mental manifestations. He referred to the paper read by Despine before the Academy of Paris, to which a prize was awarded, in which insanity was described as sickness of the soul, and in which the declaration was made that physical disease should not be associated with the idea of insanity. Dr. Gray characterized this so-called insanity of Despine as simply sin, when he speaks of passions dominating and overwhelming the individual. Dr. G. also referred to Dr. Ray in his Jurisprudence of Insanity as advocating moral insanity almost entirely on historical cases, and thought that it was a very significant fact that a superintendent with Dr. Ray's very large experience should have drawn his conclusions as to the existence of such a myth from cases that had been handed down in the books, instead of giving cases from his own clinical observation. He thought much of the difficulty with regard to moral insanity had arisen from the inconsiderable mingling of cases of arrested development, and moral delinquency with disease that they presented entirely different cases; while this class were delinquent morally and intellectually from hereditary influences and association with early degrading habits, they were not the subjects of disease, and therefore not lunatics in any true medical sense; that our present civilization recognized this and provided homes, suitable education and reformatory institutions for their development and care, and for the protection of society from their vicious and unrestrained passions, and for the higher class of delinquents mentioned by Despine, penitentiaries and work-houses.

It is satisfactory to find that Dr. Ray's opinion has been modified, and he had no doubt that if Dr. Brigham had tried, in this age of increased clinical observation, he would have reversed his judgment on this point. Dr. Gray stated that, when he first commenced the study of insanity from reading, he fully believed in the existence of moral insanity, but when he came to apply the test of clinical examination, failed to find any such cases as were described in the books; that some of these cases proved to be only deep ingrained immorality, while others were only cases of general insanity, with moral perversion strongly marked, at any rate, in all the cases he had seen, both ordinary and criminal, he had never met a case of moral insanity. He believed, with a recent English writer, that such cases had their existence only in ink and paper.

The paper of Dr. Patterson was a very able and lucid exposition of the subject, it was further discussed by Dr. Compton, of Mississippi, Drs. Knight and Seguin, of New York, and Dr. Buck, of Canada; Drs. Seguin and Buck maintaining the affirmative.

The next paper was read by Dr. Buckham, of Michigan, on "Medical Testimony, with Special Reference to Cases of Insanity." In it, Dr. B. showed that general medical practitioners were not properly considered "experts" in insanity, because in an ordinary medical education, mental diseases are not taught. He then passed to the question of emotional insanity, and took the ground that such a state of mind had never been proved, and his easy inference was that it had no existence in fact. The paper closed with the authors' suggestions for changes in medical educations, to remove the evils of which he had spoken.

A committee, consisting of Drs. Gray and Knight, of New York, and Hildreth, of West Virginia, was then appointed to consider the suggestions of Dr. Buckham, and the subject of medical expert testimony in general, and report to the Association at its next session.

